# Balticolid: A New 12-Membered Macrolide with Antiviral Activity from an *Ascomycetous* Fungus of Marine Origin

**DOI:** 10.3390/md9050844

**Published:** 2011-05-13

**Authors:** Muftah A. M. Shushni, Rajinder Singh, Renate Mentel, Ulrike Lindequist

**Affiliations:** 1 Department of Pharmacognosy, Faculty of Pharmacy, Garyounis University, P.O. Box 5341, Benghazi, Libya; E-Mail: dr.rajindersingh@yahoo.com (R.S.); 2 Institute of Pharmacy, Ernst-Moritz-Arndt-University, Friedrich-Ludwig-Jahn-Strasse 17, D-17487 Greifswald, Germany; E-Mail: renate.mentel@uni-greifswald.de (R.M.); 3 Friedrich-Loffler-Institute of Medical Microbiology, Ernst-Moritz-Arndt-University, Martin-Luther-Strasse 6, D-17487 Greifswald, Germany; E-Mail: lindequi@uni-greifswald.de (U.L.)

**Keywords:** balticolid, antiviral, herpes simplex virus, marine fungi

## Abstract

A new 12-membered macrolide, balticolid (**1**) was isolated from the EtOAc extract of the culture broth of fungal strain 222 belonging to the Ascomycota, which was found on driftwood collected from the coast of the Greifswalder Bodden, Baltic Sea, Germany. The structure of balticolid was determined to be (3*R*,11*R*), (4*E*,8*E*)-3-hydroxy-11-methyloxacyclododeca-4,8-diene-1,7-dione using extensive spectral data as well as the modified Mosher ester method. Balticolid (**1**) displayed anti-HSV-1 activity with an IC_50_ value of 0.45 μM.

## 1. Introduction

Marine microorganisms are recognized as important sources of pharmacologically active metabolites [1–3]. In particular, a growing number of marine-derived fungi have been reported to produce novel bioactive secondary metabolites. In an earlier part of this chemical investigation we described the isolation and structure elucidation of several new antiviral naphthalenone derivatives from the EtOAc extract of an ascomycetous fungus [4]. On further investigation of this fungus, we describe herein the isolation, structure elucidation and the biological activity of the novel 12-membered macrolide, balticolid (**1**).

Fungal metabolites that contain twelve-membered lactone rings like recifeiolide from *Cephalosporium recifei* [5], cladospolides A-C from *Cladosporium cladosporioides* [6] and *Cladosporium tenuissimum* [7], patulolides A-C from *Penicillium urticae* [8,9], pandangolides 1–4 from *Cladosporium herbarum* [10] and chloriolide from *Chloridium virescens* var. *chlamydosporum* [11] have already been reported.

## 2. Results and Discussion

Balticolid (**1**) was obtained as colorless oil. Its molecular formula was determined to be C_12_H_16_O_4_ by ESIMS at *m/z* 225 [M + H]^+^ and by HRESIMS at *m/z* 471.2001 for C_24_H_32_O_8_Na as [2M + Na]^+^, suggesting the existence of five degrees of unsaturation. The UV spectrum showed absorption bands consistent with a conjugated carbonyl group. Inspection of the ^1^H and ^13^C NMR data **(**Table 1) together with DEPT and HMQC spectral data revealed the presence of one methyl, three methylenes, six methines (four olefinic) and two quaternary carbons (carbonylic).

**Figure 1.**
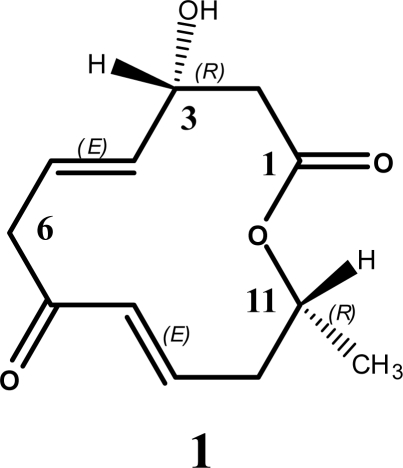
Structure of balticolid (**1**).

In addition, the IR spectrum showed the presence of a hydroxyl group (3435 cm^−1^) and *trans* disubstituted alkenes (1662 cm^−1^). An absorption band appeared at 1716 cm^−1^, and is due to the conjugated ketone, the absorption band of the 12-membered lactone potentially merged with the aforementioned as a single absorption band. The ^1^H NMR splitting pattern of **1** showed coupling between the protons at *δ*_H_ 1.34 and 5.11 which indicates the presence of a CH_3_-CH-O group. The ^1^H-^1^H COSY and ROESY spectra revealed two structural elements, one of C-2 to C-6 and the second from the unsaturated methine carbon C-8 to methyl group C-12. HMBC correlations observed from methylene protons H_2_-2 (*δ*_H_ 2.68, 2.62) and deshielded oxymethine proton H-11(*δ*_H_ 5.11) to carboxy carbon C-1 established the location of the ester linkage (Table 1). The HMBC correlation of H-3 (*δ*_H_ 4.54) with C-1, C-2 together with molecular formula C_12_H_16_O_4_ indicated the presence of a methine group and a hydroxyl group at C-3. The presence of a methyl group at C-11 was deduced by the HMBC correlation of H_3_-12 (*δ*_H_ 1.34) with C-9 (*δ*_C_ 148.1), C-10 (*δ*_C_ 39.6) and C-11 (*δ*_C_ 72.1). The geometries of C4-C5 and C8-C9 double bonds were assigned *E*, *E* on the basis of ^1^H-^1^H coupling constants (*J*_H4-H5_ = 15.9 Hz, *J*_H8-H9_ = 16.1 Hz respectively) leading to the gross structure of **1** as shown (Figure 1). Therefore, compound **1** was established as (4*E*,8*E*)-3-hydroxy-11-methyloxacyclododeca-4,8-diene-1,7-dione and named as balticolid.

**Figure 2.**
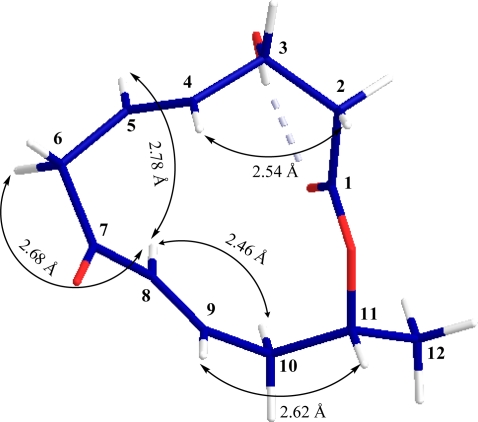
Key NOEs observed for balticolid (**1**), and their corresponding interatomic distances (Å).

The orientation of hydroxyl group at C-3 (*δ*_H_ 69.1) was assigned by taking into account the ^1^H-^1^H coupling constant values of H-3 (*δ*_H_ 1.34) with all the neighboring protons (*J*_H2a-H3_ = 3.4 Hz, *J*_H2b-H3_ = 4.7 Hz, *J*_H3-H4_ = 2.8 Hz). On the basis of vicinal coupling constant values for other protons a 3D structure for **1** was constructed as shown in Figure 2. These findings were also supported by cross peaks in ROESY spectrum and difference NOEs experiments.

**Figure 3.**
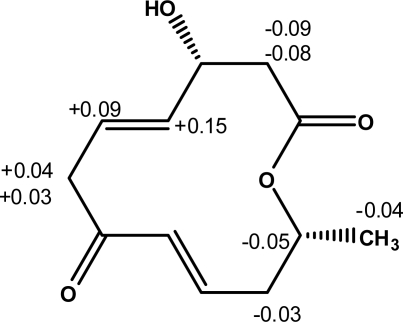
Key ^1^H NMR chemical shift differences Δδ (*δ_S_* – *δ_R_*) in ppm for the MTPA esters of **1**.

Furthermore, the absolute configuration of balticolid (**1)** at C-3 was deduced by modified high-field ^1^H NMR Mosher method [12–14]. In order to determine the absolute configuration at C-3, balticolid (**1**) was treated with (*R*) and (*S*)-α-methoxy-α-trifluoromethyl-phenylacetyl chloride (MTPA-Cl) in the presence of 4-dimethylaminopyridine (DMAP) and dicyclohexylcarbodiimide to give the (*S*)-and (*R*)-MTPA esters respectively. In the ^1^H NMR spectrum of the (*S*)-MTPA ester, the proton signals assigned to H-2a, and H-2b were observed at a higher field than those of the (*R*)-MTPA ester, and H-4, H-5, H-6a and H-6b were observed at a lower field than those of the (*R*)-MTPA ester. The bulk effect on chemical shifts induced by esterification with the chiral reagents was calculated using Δ*δ* = (*δ_S_* – *δ_R_*), where *δ_S_* and *δ_R_* are the shifts (in ppm) of diagnostic protons neighboring the chiral center in balticolid (**1**) of the (*S*) and (*R*) Mosher’s esters respectively. The absolute configuration of **1** was obtained by positioning the protons with positive Δ*δ* values on the right side and those with negative Δ*δ* values on the left side in the model described by Ohtani *et al.* [13] which clearly showed that the absolute configuration at C-3 was *R* (Figure 3).

The relative configuration of **1** at C-11 was elucidated from the interactions observed in difference NOEs experiments. By applying a decoupling field to methine proton H-8 (*δ*_H_ 5.99) there was enhancement of H-5 (*δ*_H_ 5.75), H-6a (*δ*_H_ 3.43) and H-10a (*δ*_H_ 2.52) indicating that these protons are situated on the same face while H-6b is on the opposite face. Furthermore, enhancement of proton H-4 (*δ*_H_ 5.73) and H-11 (*δ*_H_ 5.11) upon irradiation of H-2b (*δ*_H_ 2.68) and H-9 (*δ*_H_ 6.78) were observed respectively (Figure 2) indicating that these protons are situated on the opposite face or β-oriented in **1**. Therefore, the absolute configurations of both chiral centers of **1** were assigned as 3*R*, 11*R*.

Compound **1** was tested at non-cytotoxic concentrations for antiviral activity against influenza A virus and Herpes simplex virus (HSV) type I. Balticolid was only found to exhibit inhibitory activity against herpes simplex virus with an IC_50_ value of 0.45 μM compared to 0.44 μM/aciclovir. The compound showed no remarkable antimicrobial activity against *Staphylococcus aureus*, *Escherichia coli*, and *Candida maltosa* at 200 μg/disc in the agar-diffusion assay. Further structural modifications to optimize physico-chemical properties, by total and semi-synthetic approach could result in more potent molecules with desirable pharmacokinetic properties. Thorough structure activity relationship (SAR) studies of balticolid derivatives might result in new drug-lead candidates which can be exploited as future drugs.

## 3. Experimental Section

### 3.1. General Experimental Procedures

TLC: silica gel 60 F_254_ on aluminum foil (Merck); detection under daylight and UV light (λ = 254 and 366 nm), and anisaldehyde (1% in a solution of 10 mL of AcOH in 10 mL of a 15% methanolic H_2_SO_4_). Column Chromatography (CC): System 1: silica gel (0.063–0.200 μm), solvent gradient: EtOAc/hexane/MeOH 65:35:5, EtOAc/MeOH 95:5, EtOAc/MeOH 50:50, and MeOH. System 2: silica gel (0.015–0.040 μm), solvent gradient: DCM/EtOAc 75:25, and EtOAc. RP-HPLC: 250 × 4 mm; Waters Xterra-RP-C18, 5 μm, gradient 10% MeOH to 100% MeOH in 15 min, 1.0 mL/min. Optical rotation and UV spectra were measured in UV MeOH [Uvasol (Merck)] on a Polarimeter MC 241 (Perkin Elmer) and a UV-2102 PC UV-VIS scanning spectrophotometer, respectively in nm λ_max_ (logε). IR spectra were measured on a Nicolet 20 DXB FT-IR spectrometer. For NMR spectroscopy the samples were dissolved in 99.95% CD_3_OD. NMR spectra were recorded at 300 K on Bruker DPX300, DMX600 NMR spectrometers locked to the major resonance of CD_3_OD. Chemical shifts are given relative to the residual solvent signal, CD_3_OD (^1^H: 3.31 ppm; ^13^C: 49.15 ppm), *δ* in ppm, *J* in Hz. MS: HPLC/MS: 1200 Series HPLC system (Agilent) coupled to a DAD-UV detector (Agilent) and an API 2000 (Sciex); in *m/z* (rel.%); HR-MS: with an ultra-performance LC system (Accela; Thermo-Fisher, Germany) coupled via automated chip-based nanoelectrospray (NanoMate; Advion, UK) to the LTQ-Orbitrap mass spectrometer (Thermo-Fisher).

### 3.2. Strain and Fermentation

The strain 222 was isolated from driftwood collected in November 2002 from the coast of Greifswalder Bodden, Baltic Sea, Germany, by G. Mernitz and B. Cuypers. The material was classified by A. W. A. M. de Cock, Centraalbureau voor Schimmelcultures, Fungal Biodiversity Centre, Utrecht, The Netherlands, and M. Unterseher, University Greifswald, Institute for Botany and Landscape Ecology, Greifswald, Germany, as a member of the order Pleosporales, Ascomycota, by ITS sequence (accession number in the European Nucleotide Archive FR852578). Since the isolate remained sterile in subcultures on different media an exact taxonomic determination was not possible. The culture has been deposited in the culture collection of the Department of Pharmaceutical Biology, University of Greifswald (Voucher number 222). Fermentation was carried out in liquid shake cultures. 500 mL Erlenmeyer flasks containing 200 mL of Hagem medium (ammonium succinate 0.5 g; KH_2_PO_4_ 0.5 g; MgSO_4_ 7H_2_O 0.5 g; FeCl_3_ (1%) 0.5 mL; glucose 5 g; malt extract 5.0 g; aqua purificata 1000 mL, pH 7.5) were inoculated with a homogenized pre-culture of a mycelial culture of the strain 222 and cultivated on a rotary shaker (125 rpm) for 19 days at room temperature.

### 3.3. Extraction and Isolation

Mycelium and fermentation broth were separated and the culture broth was extracted with EtOAc. The organic layer was dried with anhydrous sodium sulfate and then concentrated in vacuum using a rotary evaporator to give a brown residue (500 mg from 11 L of culture broth). The extract was fractionated by CC (system 1) to yield seven fractions (A–G) according to chemical monitoring by TLC. Fraction D (70 mg) was further subjected to CC (system 2) and 8 fractions (D1–D8) were separated through TLC guided fractionation. Fraction D4 was further purified by RP-HPLC to yield 6.5 mg of balticolid (**1**).

### 3.4. Balticolid (**1**)

Colorless oil, [α]^22^d + 135.2 (c = 0.35, MeOH); TLC EtOAc:DCM (3:1) R_f_ = 0.3 (yellow spot after spraying with anisaldehyde/H_2_SO_4_ reagent and heating); HPLC UV detection at 216 nm, R_t_ = 12.2 min.; UV in MeOH λ_max_ (log ε) 214 (5456/3.736); IR (KBr) υ_max_ 3435, 1715, 1662, 1170, 980 cm^−1^; HPLC-ESI-MS (ammonium acetate buffer): +ESI *m/z* 225 [M + H]^+^, 242 [M + NH_4_]^+^, 247 [M + Na]^+^ and 470 [2M + Na]^+^; –ESI m/z 223 [M – H]^−^. HR-(+)-ESI-MS: 471.2001, calcd. 471.1995 for C_24_H_32_O_8_Na as [2M + Na]^+^.

### 3.5. Preparation of (*S*)- and (*R*)-MTPA Esters of Balticolid (**1**)

Two portions (1.5 mg each) of **1** were dissolved in dry CH_2_Cl_2_ (1 mL) containing 1 mg of dimethylaminopyridine and eight equivalents of dicyclohexylcarbodiimide. Six equivalents of either the (*S*) or the (*R*) isomer of α-methoxy-α-trifluoromethylphenylacetyl chloride were added to a portion of **1**. The reaction mixtures were allowed to stand for 24 h at room temperature. The reaction was quenched by addition of 1.0 mL of water, and the mixture was subsequently extracted with Et_2_O (3 × 1.0 mL). The Et_2_O soluble layers were combined, dried over anhydrous MgSO_4_ and evaporated. The residue was subjected to column chromatography over silica gel using *n*-hexane-EtOAc (13:1) to yield the (*S*)-MTPA and (*R*)-MTPA esters. The key ^1^H NMR chemical shift differences Δ*δ* (*δ_S_* *– δ_R_*) in ppm for the MTPA esters of **1** are shown in Figure 3.

### 3.6. Biological Activities

An agar diffusion assay according to the European Pharmacopoeia [12] was used to determine antibacterial and antifungal activity. Ampicillin was used as positive control. The following test strains were used: *Staphylococcus aureus* ATCC 6538, *Escherichia coli* ATCC 11229, and *Candida maltosa* SBUG 700. The compound was tested for antiviral activity against influenza A virus and Herpes simplex virus (HSV) type I using a test system as described by Shushni *et al*. [4]. Briefly, the virus-host systems influenza A virus—Madine-darby canine kidney (MDCK) cells and Herpes simplex virus—African green monkey kidney (Vero) cells were used. Confluent monolayers of the cells were pre-incubated treated with different concentrations of balticolid or reference compounds amantadine and acyclovir in four replicates for 30 min at 37 °C. MDCK cells were infected with 30 TCID_50_ (tissue culture infectious dose leading to virus infection of 50% of the cells) of influenza virus A and Vero cells with 30 TCID_50_ of HSV-I and further incubated for 72 h at 37 °C. The antiviral effect of test compounds was determined by neutral red assay in comparison to infected cells without test compound and non infected cells. Before antiviral tests a possible cytotoxicity of balticolid for MDCK and Vero cells was measured also by the neutral red assay. Only non-cytotoxic concentrations were used for the antiviral tests.

## Figures and Tables

**Table 1.** ^1^H [CD_3_OD, 600 MHz] and ^13^C NMR [CD_3_OD, 150 MHz] spectral data for compound **1**^a^.

**Position**	***δ*_H_^b^**	**^1^H-^1^H COSY**	**ROESY**	***δ*_C_^c^**	**HMBC (H→C)**
1	-	-	-	172.0 (s)	-
2	2.62 (1H, dd, 13.4, 3.4 Ha)	2b, 3	-	43.3 (t)	1, 3, 4
	2.68 (1H, dd, 13.4, 4.7 Hb)	2a, 3	5/4, 11		1, 3, 4, 5
3	4.54 (1H, m)	4, 2a/b (6a)	6a/6b	69.1(d)	1, 2
4	5.73 (1H, dd, 15.9, 2.8)	5, 3	9, 2a/2b	138.4 (d)	3, 5, 6
5	5.75 (1H, m)	4, 6a/b	2a/2b	125.2 (d)	3, 4, 6, 7
6	3.43 (1H, br dd, 13.6, 6.8, Ha)	6b, 5, 3	8	45.8 (t)	4, 5, 7, 8
	3.22 (1H, ddd 13.6, 4.5, 1.7, Hb)	6a, 5	9, 3		4, 5, 7
7	-	-	-	202.7 (s)	-
8	5.99 (1H, br d, 16.1)	9, 10a/b	10a/10b	132.8 (d)	6, 7, 9, 10, 11
9	6.78 (1H, ddd, 16.1, 8.9, 6.6)	8, 10a/b	6b/6a, 5/4, 11, 10b/10a	148.1 (d)	8, 10, 11
10	2.52 (1H, dddd, 13.4, 6.6, 3.3, 1.3, Ha)	10b, 9, 11 (8)	8, 11	39.6 (t)	8, 9, 11
	2.38 (1H, ddd, 13.4, 11.0, 8.9, Hb)	10a, 9, 11, (8)	8, 11		8, 9, 11, 12
11	5.11 (1H, ddq, 11.0, 3.3, 6.3)	10a/b, 12	10b/10a, 9	72.1 (d)	1, 9, 10, 12
12	1.34 (3H, d, 6.3)	11	10b/10a, 2b/2a	21.1 (q)	9, 10, 11

a All assignments are based on 1D and 2D measurements (DEPT, COSY, ROESY, HMQC, HMBC).

b Chemical shifts (*δ*) were expressed in ppm, *J* in Hz.

c Implied multiplicities were determined by DEPT (C = s, CH = d, CH_2_ = t).
